# Low 5HT_1B_ and 5HT_4_ Receptor Neurotransmission as a Potential Link Between Cholesterol Metabolism and Suicide Risk

**DOI:** 10.1111/jnc.70466

**Published:** 2026-05-13

**Authors:** Hans O. Kalkman, Lukasz Smigielski

**Affiliations:** ^1^ Child and Adolescent Psychiatry and Psychotherapy Psychiatric University Hospital Zurich, University of Zurich Zurich Switzerland

**Keywords:** ceramide, cholesterol, IDO, IL‐6, impulsivity, lipid raft, S100A10, tryptophan

## Abstract

Elevated levels of the inflammatory cytokine IL‐6 and decreased levels of cholesterol in blood and brain tissue have been reported in studies of individuals who attempted or completed suicide. The mechanisms underlying these effects remain unclear. In this review, we discuss a potential mechanistic link between these observations involving lipid raft function and serotonergic signaling. Reduced cholesterol availability may affect lipid raft function and could, potentially through reduced levels of the lipid raft protein S100A10 (p11), result in diminished cell‐surface expression of the serotonin receptors 5‐HT_1B_ and 5‐HT_4_. Both receptors have been implicated in the suppression of impulsive and aggressive behavior. Lipid rafts are also organizing platforms for GABA, glutamate, and serotonin transporters. Reduced serotonin reuptake could contribute to the often‐reported decrease in the serotonin metabolite 5‐hydroxyindoleacetic acid (5‐HIAA) in suicidal individuals. Inflammatory cytokines, including IL‐6, may further influence serotonergic signaling by increasing expression of the enzyme indoleamine‐2,3‐dioxygenase (IDO), which enhances tryptophan degradation through the kynurenine pathway. Reduced tryptophan availability may limit serotonin synthesis and thereby decrease activation of the 5‐HT_1B_ and 5‐HT_4_ receptors. These observations suggest that the combined effects of low cholesterol, elevated IL‐6 signaling, and reduced tryptophan availability may increase impulsivity and thereby heighten vulnerability to suicidal behavior. This framework accommodates findings from genetic and biomarker studies in suicidal patients. Owing to its effect on lipid raft organization, the fish‐oil component docosahexaenoic acid (DHA) might modulate these processes and potentially reduce suicide risk in individuals with low cholesterol levels.

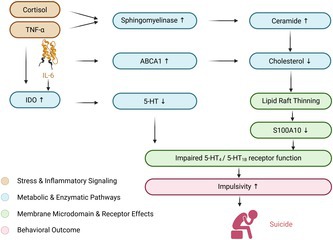

## Graphical abstract

Stress and inflammatory signals (e.g., IL‐6, TNFα, cortisol) may disrupt cholesterol metabolism and membrane microdomains known as lipid rafts. This can impair the function of serotonin receptors (5‐HT1_B_ and 5‐HT_4_) that regulate impulsive behavior. In parallel, inflammation may reduce serotonin (5‐HT) availability by activating indoleamine‐2,3‐dioxygenase (IDO), which increases tryptophan degradation. Together, these pathways may increase impulsivity and vulnerability to suicidal behavior.

**Abbreviations**
5‐HIAA: 5‐hydroxyindoleacetic acid
5‐HT: 5‐hydroxytryptamine (serotonin)
5‐HT1_B_
: 5‐hydroxytryptamine receptor 1B
5‐HT_4_
: 5‐hydroxytryptamine receptor 4
8‐OHdG: 8‐hydroxydeoxyguanosine
ABCA1: ATP‐binding cassette transporter A1
ADHD: attention‐deficit/hyperactivity disorder
APP: amyloid precursor protein
AT1 receptor: angiotensin II type 1 receptor
Aβ: amyloid beta
Aβ40: amyloid beta 40
Aβ42: amyloid beta 42
BACE: β‐secretase
BBB: blood–brain barrier
C99: 99‐amino‐acid C‐terminal fragment of amyloid precursor protein
CoQ10: coenzyme Q10
CRP: C‐reactive protein
CSF: cerebrospinal fluid
DHA: docosahexaenoic acid
DNA: deoxyribonucleic acid
GABA: gamma‐aminobutyric acid
gp130: glycoprotein 130
IDO: indoleamine 2,3‐dioxygenase
IL‐1β: interleukin‐1 beta
IL‐6: interleukin‐6
JAK: Janus kinase
MAO: monoamine oxidase
MAO‐A: monoamine oxidase A
MAO‐B: monoamine oxidase B
MDD: major depressive disorder
NMDA: N‐methyl‐D‐aspartate
NRF2: nuclear factor erythroid 2‐related factor 2
p11: S100A10
PUFAs: polyunsaturated fatty acids
QA: quinolinic acid
ROS: reactive oxygen species
sIL‐6R: soluble interleukin‐6 receptor
SNPs: single‐nucleotide polymorphisms
STAT3: signal transducer and activator of transcription 3
TNFR1: tumor necrosis factor receptor 1
TNFα: tumor necrosis factor alpha
TPH2: tryptophan hydroxylase 2

## 1 Introduction

Suicide is frequently associated with depression (Cai et al. 2021; Grossberg and Rice 2023), but suicide risk is also increased in several other psychiatric and neurological disorders (Bachmann 2018; Bolton and Robinson 2010). In addition, certain phenotypic traits, such as alexithymia, characterized by difficulties in identifying and describing emotions, may further increase vulnerability to suicidality. Indeed, alexithymia has been associated with greater disorder severity and higher levels of suicidal ideation, particularly in trauma‐related conditions (De Berardis et al. 2020). In addition, the COVID‐19 pandemic has acted as a major socio‐environmental stressor, increasing psychiatric vulnerability through social disconnection and psychosocial burden. These factors have been associated with negative emotional states such as loneliness, hopelessness, despair and increased suicide risk, particularly among vulnerable subgroups, including younger and elderly individuals (Amerio et al. 2020). Numerous biological factors have been identified that may contribute to suicidal behavior (Capuzzi et al. 2020; Johnston et al. 2022; Marini et al. 2016). These include stress‐related parameters, such as elevated cortisol levels and non‐suppression in the dexamethasone suppression test (Kim et al. 2023; Stanley et al. 2019), inflammation markers (such as increased CRP and pro‐inflammatory cytokines, including IL‐6, TNFα, and IL‐1β) (Neupane et al. 2023; Serafini et al. 2020), alterations in monoaminergic neurotransmitters and their MAO‐derived metabolites (Galfalvy et al. 2009; Hoertel et al. 2021; Johnston et al. 2022), and reduced cholesterol levels (Johnston et al. 2022; Kim et al. 2023). Most of these factors are also associated with depression (Iwata et al. 2013; Mansur et al. 2015; Schmidt et al. 2011). One notable exception may be cholesterol metabolism. This review examines the possibility that altered cholesterol homeostasis, impaired lipid raft function, and downstream effects on serotonergic signaling contribute to impulsivity and suicidal behavior.

## 2 Cholesterol Metabolism in Depression and Suicide

### 2.1 High Cholesterol in Depression but Low Cholesterol in Suicide

Cholesterol levels are often elevated in depression (Wagner et al. 2019) (but see Bharti et al. 2021), especially in patients with hypersomnic “atypical” depression (de Kluiver et al. 2023; van der Heijden and Houben 2023). In contrast, cholesterol levels in suicidal individuals have frequently been reported to be decreased in serum (Aguglia et al. 2019; Suneson et al. 2019; Wu et al. 2016) and, according to some research, also in brain tissue (Lalovic et al. 2007). It should be noted, however, that a substantial number of studies do not confirm a decrease in serum cholesterol levels in suicidal individuals (Capuzzi et al. 2018; Tanskanen et al. 2000; Zhao et al. 2020).

### 2.2 Pharmacokinetic Profile of Cholesterol

Cholesterol does not cross the blood–brain barrier (BBB) to an appreciable extent (Dietschy 2009), and serum cholesterol levels therefore do not necessarily reflect cholesterol levels in the brain (Schreurs 2010). In the adult brain, cholesterol is synthesized in the endoplasmic reticulum of astrocytes (Zhemkov et al. 2021) and subsequently packed into apolipoprotein E‐containing lipoprotein particles for distribution via the cerebrospinal fluid (CSF) to other brain cells (Schreurs 2010). This process requires the cholesterol carrier ABCA1 (Schreurs 2010). ABCA1 functionality has been reported to influence suicide risk, whereby increased cholesterol efflux may increase the risk (Knowles et al. 2018). These features of brain cholesterol handling raise the possibility that altered cholesterol trafficking may affect membrane microdomains rather than simply total cholesterol levels.

## 3 Lipid Raft Biology and Membrane Organization

### 3.1 Cholesterol as a Structural Component of Lipid Rafts

Together with sphingolipids and phospholipids, cholesterol increases membrane ordering and contributes to the formation of gel‐like membrane microdomains, commonly referred to as “lipid rafts” within the phospholipid bilayer (Tracey et al. 2018; Zhemkov et al. 2021). Cholesterol‐rich phospholipid mixtures not only exhibit a more ordered acyl‐chain structure but are also thicker than normal lipid bilayers and represent anchoring sites for numerous receptors and ion channels (Eyster 2007; Simons and Toomre 2000). A molecule that has been proposed to interact with cholesterol and influence lipid raft formation is the amyloid precursor protein (APP) fragment C99 (Area‐Gomez and Schon 2024). This 99‐amino‐acid protein is generated from APP by the enzymatic activity of β‐secretase (BACE) (Evin et al. 2003). C99 carries a cholesterol‐binding site and has been suggested to participate in cholesterol trafficking (Area‐Gomez and Schon 2024; Pera et al. 2017). The levels of C99 are regulated by the protease γ‐secretase (Schreiner et al. 2015). When γ‐secretase cleaves C99 to generate Aβ40, the cholesterol‐binding region is removed (Area‐Gomez and Schon 2024). The sequential activity of BACE and γ‐secretase therefore regulates C99 levels and may influence the cholesterol content of lipid rafts, including those located at mitochondrial membranes. γ‐Secretase localizes to lipid raft domains associated with mitochondria (Zhemkov et al. 2021). A reduction in membrane thickness owing to decreased cholesterol content may cause a slight tilting of the enzyme (Area‐Gomez and Schon 2024). This may have profound functional consequences, as the proteolysis of C99 can result in the formation of an Aβ‐molecule with a slightly different length, Aβ42. The Aβ40/Aβ42 ratio has therefore been proposed as a surrogate marker for membrane thickness of lipid rafts (Area‐Gomez and Schon 2024).

### 3.2 Sphingomyelin and Ceramide Dynamics in Lipid Rafts

Sphingomyelin is another important constituent of cell membranes, myelin sheaths, and lipid rafts (Eyster 2007; Goñi 2022). Sphingomyelins consist of a fatty amino alcohol (mostly sphingosine), a long‐chain fatty acid (chain lengths usually ranging from C_18_ to C_22_), and phosphorylcholine (Custodia et al. 2022). Hydrolytic removal of phosphorylcholine by sphingomyelinase enzymes gives rise to ceramides (Cutler and Mattson 2001). Ceramides with fatty acid chains between C_16_ and C_22_ represent an important group of bioactive lipids (Field et al. 2020; Kong et al. 2018; Kurz et al. 2019). These ceramides can displace cholesterol from lipid rafts (Megha and London 2004; Silva et al. 2009; Yu et al. 2005) and promote the clustering of pro‐apoptotic molecules (Gulbins et al. 2015). Because ceramides can be effectively transferred between membranes (Clausmeyer and Fröhlich 2023; Roszczyc‐Owsiejczuk and Zabielski 2021), they may affect lipid raft organization throughout the cell (Mignard et al. 2020). Ceramides have been shown to decrease mitochondrial respiratory chain activity, thereby increasing reactive oxygen species (ROS) production and oxidative stress. Furthermore, ceramides can promote permeabilization of the mitochondrial outer membrane and reduce mitochondrial membrane potential, processes that may ultimately lead to mitophagy and apoptosis (Kogot‐Levin and Saada 2014). TNFα acting via the TNFR1 receptor (Martinez et al. 2012; Singh et al. 1998; Xu et al. 1998), as well as stimulation of glucocorticoid (Cifone et al. 1999; Quintans et al. 1994) and mineralocorticoid receptors (Zhang et al. 2016), can induce ceramide production through the hydrolysis of sphingomyelin by sphingomyelinases (for review see Kalkman and Smigielski 2025). These observations suggest that chronic stress and inflammation may deteriorate lipid raft function. High CSF TNFα levels have been associated with suicidal ideation (Juengst et al. 2014; Lindqvist et al. 2011). The data on cortisol levels in individuals who have attempted suicide are, however, mixed, with reports of increases, decreases, or no significant change (Kang et al. 2023). Such changes in lipid raft composition may have important consequences for membrane proteins involved in serotonergic signaling.

## 4 Lipid Raft Dysfunction and Serotonergic Signaling

### 4.1 S100A10 as an Anchor for Serotonin Receptors

The lipid raft protein S100A10 (Benaud et al. 2003) is a multifunctional protein that forms a heterotetrameric complex with Annexin A2. This complex interacts with several ion channels and regulates their cellular localization and function (Seo and Svenningsson 2020). Notably, S100A10 (also known as p11) interacts with two serotonin receptors, i.e., the 5‐HT_1B_ and 5‐HT_4_ receptors, and is required for their cell‐surface expression and functional responses (Svenningsson et al. 2013; Warner‐Schmidt et al. 2009). Reductions in S100A10 mRNA and protein levels have been reported in the hippocampus, amygdala, prefrontal cortex, and peripheral blood mononuclear cells of suicide victims (Svenningsson et al. 2013).

### 4.2 5‐HT Receptor Dysfunction and Impulsivity

Stimulation of 5‐HT_1B_ (Nautiyal et al. 2015) and 5‐HT_4_ receptors (Hori et al. 2024) reduces impulsivity, whereas conversely, a reduction in signaling increases impulsive responding. A loss‐of‐function mutation in the promoter region of the 5‐HT_1B_ receptor has been observed in individuals who have attempted suicide (Huang et al. 1999; Murphy et al. 2011; Yang et al. 2021). In humans, cells of the adrenal zona glomerulosa express 5‐HT_4_ receptors, and their activation by serotonin (released from intra‐adrenal perivascular mast cells) stimulates the synthesis and release of aldosterone (Lefebvre et al. 1998; Seccia et al. 2018). Dysfunctional 5‐HT_4_ neurotransmission might therefore reduce aldosterone synthesis. Notably, low serum aldosterone levels have been reported in patients with major depressive disorder (MDD) who have attempted suicide, in contrast to MDD patients without suicide attempts (Hallberg et al. 2011). This finding is notable because aldosterone levels in blood and saliva from patients with MDD have otherwise been reported to be consistently elevated (Nowacki et al. 2020 and references cited therein). It should be noted, however, that angiotensin II acting via the AT1 receptor represents the major determinant of aldosterone synthesis rather than 5‐HT4 signaling. The AT1 receptor is also localized in lipid rafts, and its function is strongly suppressed by cholesterol depletion (Adebiyi et al. 2014). It is therefore possible that low aldosterone levels could represent a peripheral biomarker of impaired lipid raft function (and thus altered 5‐HT_4_ neurotransmission) in suicidal individuals.

## 5 Inflammatory Mechanisms Associated With Suicide

### 5.1 IL‐6 Elevation in Disorders Associated With Suicide

Elevated IL‐6 levels in blood are considered a state marker of depression, as they are increased during depressive episodes (Kern et al. 2014; Haapakoski et al. 2015; Sasayama et al. 2013) but return to normal values after a successful antidepressant treatment (Gay et al. 2021; Strawbridge et al. 2015; Więdłocha et al. 2018; Zhan et al. 2020). IL‐6 levels are also increased in blood samples of patients with bipolar disorder, schizophrenia, panic disorder, ADHD, Tourette syndrome, autism, migraine with aura, temporal lobe epilepsy, amyotrophic lateral sclerosis, and restless legs syndrome (Table 1). One of the most consistent findings in studies that measured cytokines in individuals who attempted or completed suicide is elevated IL‐6 levels. This has been observed in measurements of CSF, blood, and postmortem brain tissue (Black and Miller 2015; Ganança et al. 2016; Jiang et al. 2022; Lindqvist et al. 2009; Neupane et al. 2023). In many of the disorders characterized by elevated IL‐6 levels, there is also evidence for increased risk of suicide (Table 1). These observations suggest that IL‐6 may be involved in the pathophysiology of suicidal behavior. However, it remains unclear whether IL‐6 plays a causal role, and if so, through what mechanism.

**TABLE 1:** Conditions associated with increased suicide risk and elevated circulating IL‐6 levels.

Condition	Reference for increased suicide attempts or suicides	Reference for elevated IL‐6
ADHD	Man et al. (2017) and Septier et al. (2019)	Darwish et al. (2019) and Elsadek et al. (2020)
Alcohol abuse	Gradus et al. (2010) and Nock et al. (2010)	Adams et al. (2020) and Bramness et al. (2023)
Alexithymia	De Berardis et al. (2020)	Honkalampi et al. (2011) and Pedrosa Gil et al. (2007)
Amyotrophic lateral sclerosis (ALS)	Fang et al. (2008)	Wosiski‐Kuhn et al. (2019)
Autism	Lai et al. (2023)	Saghazadeh et al. (2019) and Yildirim et al. (2023)
Bipolar disorder	Nierenberg et al. (2023) and Zalsman et al. (2006)	Goldsmith et al. (2016) and Jiang et al. (2022)
Migraine with aura	Kim et al. (2025) and Wei et al. (2023)	Wang et al. (2015)
Panic disorder	Zhang et al. (2022)	Liu et al. (2021) and Zou et al. (2020)
PTSD	Bentley et al. (2016), Fox et al. (2021), and Gradus et al. (2010)	Baker et al. (2002), Gill et al. (2013), and Maes et al. (1999)
Restless legs syndrome	Chenini et al. (2022) and Zhuang et al. (2019)	Uslu et al. (2021)
Schizophrenia/first‐episode psychosis	Ayesa‐Arriola et al. (2018), Bachmann (2018), and Brown (1997)	Miller et al. (2011) and Stojanovic et al. (2014)
Temporal lobe epilepsy	Bell et al. (2009) and de Oliveira et al. (2011)	Liimatainen et al. (2009)
Tourette syndrome	Fernández de la Cruz et al. (2017)	Li et al. (2022)

### 5.2 IL‐6 Signaling, ABCA1 Activation, and Tryptophan Metabolism

Neurons and astrocytes, in contrast to cerebrovascular endothelium cells and microglia, exhibit little or no IL‐6 receptor expression and therefore these cells cannot be efficiently activated via classical IL‐6 signaling (Campbell, Erta, et al. 2014). Nevertheless, IL‐6 can activate neurons and astrocytes through a mechanism involving a soluble form of the IL‐6 receptor. This occurs after sequestration of IL‐6 by a soluble form of the IL‐6 receptor (sIL‐6R), which is proteolytically cleaved from activated microglia and cerebrovascular endothelium cells. The IL‐6/sIL‐6R complex can then activate gp130‐expressing cells, including neurons and astrocytes (Rose‐John et al. 2023). This mechanism is referred to as “trans‐signaling” (Campbell, Erta, et al. 2014; Rose‐John et al. 2023). The signal transduction pathway downstream of gp130 involves activation of the tyrosine kinase JAK, which phosphorylates the transcription factor signal transducer and activator of transcription‐3 (STAT3). STAT3 is a transcription factor that regulates gene expression in both nuclear and mitochondrial DNA (Klinge 2020).

It is possible that IL‐6 signaling influences cholesterol metabolism, although only limited evidence currently supports this notion. One report suggests that IL‐6 trans‐signaling increases expression of the cholesterol transporter ABCA1, potentially promoting cholesterol efflux (Frisdal et al. 2011). However, this mechanism alone is unlikely to explain the reduced cholesterol levels frequently reported in suicidal patients. A further consequence of IL‐6 trans‐signaling is the induction of the IDO enzyme (Kim et al. 2012; Litzenburger et al. 2014). During infections, activation of IDO in immune cells depletes tryptophan, an amino acid that is rate‐limiting for the growth of pathogens (Campbell, Charych, et al. 2014; Kita et al. 2002; MacKenzie et al. 2007). IDO converts tryptophan to kynurenine, which in microglia is further metabolized to quinolinic acid (QA) (Brundin et al. 2015; MacKenzie et al. 2007). QA is a potent agonist of NMDA‐type glutamate receptors and can be toxic to neurons and astrocytes (Braidy et al. 2009).

There is evidence for enhanced tryptophan degradation in suicidal individuals (Bradley et al. 2015; Sublette et al. 2011). Sublette et al. studied patients with MDD and observed elevated blood levels of kynurenine specifically in the subgroup of suicide attempters. In the CSF of suicide attempters, the IL‐6 and QA levels were increased, and the levels of these two markers were correlated with each other as well as with scores on the suicide‐intent scale (Erhardt et al. 2013). These CSF QA levels were independent of depression ratings (Erhardt et al. 2013). The same research group measured CSF levels repeatedly over a period of 2 years, finding that QA levels remained continuously elevated, with the highest values measured in close proximity to the suicide attempt (Bay‐Richter et al. 2015). Finally, post‐mortem studies have reported QA‐reactive microglia in the cingulate cortex of individuals who died by suicide (Steiner et al. 2011). These findings raise the question of whether suicidality is primarily related to reduced tryptophan availability or to increased levels of kynurenine metabolites. Although this question cannot yet be answered definitively, experiments in animals indicate that reduced central serotonin levels lead to increased impulsive responding (Bizot et al. 1999). Similarly, a reduction in serotonin synthesis caused by tryptophan depletion has been reported to increase aggression in aggressive humans (Bjork et al. 2000; Cleare and Bond 1995).

### 5.3 Reduced Serotonin Synthesis

Serotonin levels can also be affected by mutations in the enzyme tryptophan hydroxylase.

Numerous SNPs in the enzyme tryptophan hydroxylase‐2 (TPH2; required for 5‐HT production) have been associated with suicide (Zill et al. 2004), especially in the context of depression (de Lara et al. 2007; Gao et al. 2012; Yoon and Kim 2009), but also in bipolar disorder (Lopez et al. 2007), personality disorder (de Lara et al. 2007), eating disorders (Slof‐Op't Landt et al. 2013), alcohol use disorder (Zupanc et al. 2011), positive symptoms of schizophrenia (Gao et al. 2012), verbal aggression and impulsive behavior (Zupanc et al. 2011), and panic disorder (Gao et al. 2012). These findings suggest that decreased 5‐HT synthesis and reduced neurotransmission may contribute to disorders characterized by impaired impulse control.

### 5.4 Integrated Mechanistic Pathway for Suicidal Behavior

In this review, we propose a mechanistic framework linking altered cholesterol metabolism, lipid raft dysfunction, and impaired serotonergic signaling to increased vulnerability to suicidal behavior. Specifically, we hypothesize, based on existing but fragmented evidence, that reduced cholesterol availability may disrupt lipid raft organization, leading to decreased S100A10 (p11) levels and reduced cell‐surface expression of the 5‐HT1_B_ and 5‐HT_4_ receptors. In parallel, inflammatory processes, particularly IL‐6‐mediated activation of the IDO pathway, may reduce tryptophan availability and further impair serotonergic neurotransmission. Together, these converging pathways may increase impulsivity and thereby contribute to suicide risk.

**FIGURE 1:**
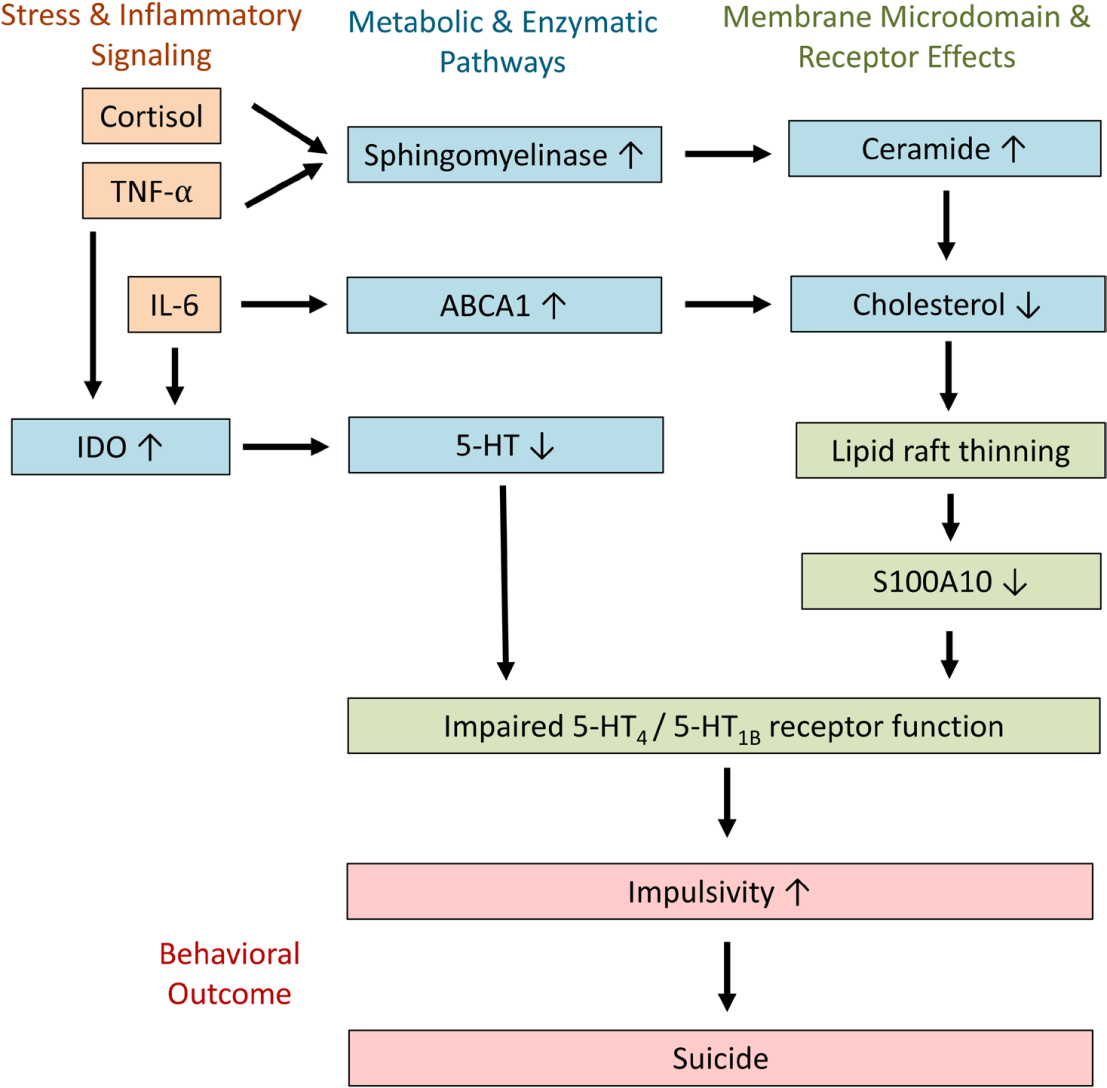
Proposed pathways linking inflammatory and metabolic risk factors to impulsivity and suicidal behavior. Impulsivity is an important risk factor for suicide. Impaired signaling through the serotonin receptors 5‐HT_4_ and 5‐HT_1B_ increases impulsive responding. Neurotransmission via these receptors can be reduced by low cholesterol levels and by increased activity of the enzyme indoleamine‐2,3‐dioxygenase (IDO), leading to reduced receptor availability and reduced agonist levels, respectively. These pathways are activated by the inflammatory cytokines TNFα and IL‐6, as well as by the stress hormone cortisol. 5‐HT, 5‐hydroxytryptamine (serotonin); ABCA1, ATP‐binding cassette transporter A1; IDO, indoleamine 2,3‐dioxygenase; IL‐6, interleukin‐6; S100A10, p11 (Ca^2+^‐binding protein of the S100 family); TNFα (tumor necrosis factor‐α).

This proposed integrative model is supported by several lines of evidence. In particular, certain risk factors for suicide may reduce cholesterol levels in lipid rafts, leading to decreased S100A10 levels, impaired anchoring of 5‐HT_1B_‐ and 5‐HT_4_ receptors, and, consequently, to less effective suppression of impulsive and aggressive behavior. Increased impulsivity could ultimately increase the likelihood of suicide attempts (Ernst et al. 2009; Isung et al. 2014; Lee et al. 2025; Stanley et al. 2019). This scenario is diagrammed in Figure 1. This framework accommodates several additional observations described in the suicide literature. A low‐cholesterol diet not only induced aggressive behavior in monkeys, but also led to significantly reduced CSF levels of 5‐hydroxyindoleacetic acid (5‐HIAA) (Kaplan et al. 1994). These findings are consistent with reports of increased violent and aggressive behavior in humans with low levels of cholesterol in blood (Repo‐Tiihonen et al. 2002; Suneson et al. 2019). Low CSF levels of 5‐HIAA have also been reported in suicide attempters (see the meta‐analysis by Hoertel et al. 2021), particularly in individuals with personality disorders (Cremniter et al. 1999), those considered highly impulsive (Cremniter et al. 1999; Spreux‐Varoquaux et al. 2001), and those who used highly lethal methods during the suicide attempt (Placidi et al. 2001). Lipid rafts at the plasma membrane are important for the anchoring and function of GABA, glutamate, and serotonin transporters (Allen et al. 2007; Magnani et al. 2004). Disruption of these membrane microdomains may reduce the transport rate of these neurotransmitters by up to 50% (Allen et al. 2007). Since monoamine oxidase‐A (MAO‐A) and monoamine oxidase‐B (MAO‐B) are localized to the outer mitochondrial membrane (Weyler et al. 1990), diminished cellular serotonin reuptake is expected to result in reduced production of the serotonin metabolite 5‐HIAA. Lipid rafts are also present on platelets (Komatsuya et al. 2020), suggesting that low cholesterol levels could affect serotonin reuptake in platelets as well. This possibility is consistent with reports of reduced platelet serotonin levels both in impulsive individuals (Dutta et al. 2017) and in impulsive suicide attempters specifically (Spreux‐Varoquaux et al. 2001), including those who used highly lethal methods (Giurgiuca et al. 2016).

Elevated IL‐6 levels have been reported in individuals with high irritability and hostility scores (Marsland et al. 2008; Sutin et al. 2010), both of which are behavioral traits associated with suicidal behavior (Jha et al. 2020; Lee et al. 2025; Orri et al. 2018). Elevated IL‐6 levels have also been reported in numerous psychiatric and neurological disorders associated with increased suicide rates (Table 1). Since suicide also occurs in individuals without clearly elevated depression scores (Nock et al. 2010), one parsimonious explanation is that impulsivity drives suicidal behavior, rather than typical depressive phenotypes, such as anhedonia, lethargy, or melancholy.

In the scheme depicted in Figure 1, two relatively independent pathways converge on impaired 5‐HT_1B_/5‐HT_4_ receptor function and increased irritability. Inflammation, reflected by elevated levels of CRP, TNFα, and IL‐1β, may impair lipid raft formation by increasing ceramide levels. Thus, inflammation and stress may contribute to suicidal behavior, although they may not represent major determinants in every individual. Tryptophan depletion represents a second major factor influencing impulsivity. This may explain why elevated CRP, TNFα, or IL‐1β levels are often, but not consistently, found to predict suicide risk (for review see Kang et al. 2023).

## 6 Oxidative Stress and Ferroptosis

### 6.1 IL‐6 Signaling and Ferroptosis

The consistent findings of elevated serum IL‐6 levels and reduced cholesterol levels in suicidal individuals provide a strong impetus to search for potential mechanisms that might link IL‐6 signaling to cholesterol depletion. Ferroptosis is an iron‐dependent form of regulated cell death that is triggered by elevated intracellular iron levels and characterized by lipid peroxidation and mitochondrial dysfunction (Chen et al. 2023; Li et al. 2020). IL‐6/JAK/STAT3‐signaling increases the transcription of hepcidin, an inhibitor of cellular iron export and therefore a potential cause of intracellular iron accumulation (Kowdley et al. 2021; Marques et al. 2009). Elevated intracellular iron levels promote the production of ROS, oxidized DNA products, such as 8‐hydroxy‐deoxyguanosine (8‐OHdG), and lipid peroxidation products derived from unsaturated fatty acids, including malondialdehyde and 4‐hydroxynonenal (Capelletti et al. 2020; Chen et al. 2023). These findings are consistent with reports of increased oxidative stress and reduced antioxidant capacity in patients with a history of suicide attempts (Odebrecht Vargas et al. 2013). Ferroptosis is counteracted by antioxidant systems, such as the transcriptional targets of nuclear factor erythroid 2‐related factor 2 (NRF2) (Esteras and Abramov 2022; Shakya et al. 2023), as well as by lipophilic antioxidants, such as coenzyme Q10 (CoQ10; ubiquinone) (Bentinger et al. 2010; Picón and Skouta 2023). CoQ10 is synthesized from mevalonate (Bentinger et al. 2010), and its production therefore competes with cholesterol synthesis within the mevalonate pathway (Picón and Skouta 2023). It is therefore conceivable that increased demand for CoQ10 could limit cholesterol synthesis. The rate‐limiting step of cholesterol synthesis downstream of the bifurcation towards CoQ10 involves squalene synthase, and the activity of this enzyme has been reported to change during ferroptosis (Picón and Skouta 2023). Genetic disruption of the NRF2 gene strongly promotes oxidative stress and ferroptosis (Ma 2013; Ye et al. 2025). Notably, mice with a genetic disruption of NRF2 (rendering them susceptible to ferroptosis and oxidative damage) display very low serum cholesterol levels (Xu et al. 2015). However, although hepcidin is expressed in the human choroid plexus and brain and is known to play a role in neuronal iron homeostasis (Vela 2018), it remains unclear whether IL‐6‐induced hepcidin transcription contributes to reduced cholesterol levels in the brain.

## 7 Biomarkers and Translational Implications

### 7.1 Circulating Biomarkers for Suicide Risk

There are two potential causes for reduced cholesterol levels in lipid rafts. First, activation of sphingomyelinase by TNFα during inflammatory processes or by glucocorticoids during stress generates ceramides that can displace cholesterol from lipid rafts. Thus, measuring ceramide levels might provide insights into these factors. Unfortunately, it is currently unknown whether ceramide levels are increased in the blood of suicidal individuals. However, ceramide levels have been reported to be elevated in blood and brain samples of patients with bipolar disorder and schizophrenia (Schwarz et al. 2008) as well as major depression (Brunkhorst‐Kanaan et al. 2019; Schumacher et al. 2022; Tomasik et al. 2016). A second potential mechanism leading to reduced cholesterol levels in lipid rafts is IL‐6‐induced ABCA1‐mediated cholesterol efflux. IL‐6 trans‐signaling can be monitored by measuring the transcription of SerpinA3 in white blood cells. SerpinA3 is a gene that appears to be selectively regulated by IL‐6 trans‐signaling rather than classical IL‐6/IL‐6R signaling (Campbell, Erta, et al. 2014; Hoffman et al. 2023). At present, there is no evidence that SerpinA3 levels are increased in the blood of suicidal individuals. However, increased expression of IL‐6 and SerpinA3 has been observed in plasma and brain samples from patients with schizophrenia (Fillman et al. 2013; Tomasik et al. 2016) and depression (Zalli et al. 2016). The combined measurement of ceramides and SerpinA3 could, in principle, capture the two major mechanisms leading to reduced lipid‐raft cholesterol levels. Given its localization within lipid rafts (Benaud et al. 2003) and its expression in peripheral blood mononuclear cells (Zhang et al. 2011), S100A10 levels could also be studied as a potential marker of lipid raft function. To capture the second pathway influencing impulsivity, these biomarkers related to cholesterol metabolism should be complemented by measurement of tryptophan levels.

### 7.2 Docosahexaenoic Acid and Lipid Raft Stabilization

The omega‐3 fatty acid docosahexaenoic acid (DHA) has been reported to increase the size of lipid rafts by enhancing segregation between the membrane bilayer and the raft domains (Rockett et al. 2012; Wassall et al. 2018). This may increase the thickness of the lipid raft membranes and improve the anchoring of proteins, thereby facilitating transport and signaling functions (Sublette 2020). Consistent with the notion that suicide risk factors may impair lipid raft function, DHA‐rich diets were reported to increase 5‐HT and 5‐HIAA levels in the mouse brain (Jiang et al. 2012). Moreover, low blood levels of DHA in medication‐free patients with depression predicted future suicide attempts (Sublette et al. 2006). Similarly, a retrospective study in U.S. military personnel found that the risk of suicide death was more than doubled in individuals with low DHA blood levels (Lewis et al. 2011). On the other hand, a very large prospective cohort study found no evidence that omega‐3 intake reduces the risk of completed suicide (Tsai et al. 2014).

There is cross‐sectional evidence that a deficiency of omega‐3 polyunsaturated fatty acids (PUFAs) is associated with hostility, aggressive behavior, and impulsivity in both psychiatric and non‐psychiatric populations (Bozzatello et al. 2020; Meyer et al. 2015), whereas supplementation with omega‐3 PUFAs has been reported to reduce symptoms of impulsivity (Marriott et al. 2016). Moreover, omega‐3 PUFA treatment of patients with severe ADHD significantly improved impulsivity, hyperactivity, and inattention scores (Sinn and Bryan 2007). A recent meta‐analysis found significant reductions in aggression following omega‐3 supplementation in multiple samples of children and adults (Raine and Brodrick 2024). Finally, a small study specifically testing DHA reported reductions in aggressive behavior and impulsivity in young males (Long and Benton 2013). Taken together, these observations suggest that, owing to its effects on lipid raft organization, DHA might contribute to the reduction or prevention of suicidal behavior in individuals with low cholesterol levels. It would therefore be of value to test whether DHA increases S100A10 levels in leukocytes and whether this response is associated with a reduction in suicide risk.

## 8 Conclusions, Limitations, and Future Perspectives

Suicidal behavior may arise from the convergence of inflammatory, metabolic, and serotonergic abnormalities. The model proposed here suggests that reduced cholesterol availability in lipid rafts, increased ceramide formation, and IL‐6‐associated tryptophan degradation may impair serotonergic mechanisms that normally suppress impulsive and aggressive behavior. This integrative perspective highlights a potential role for membrane microdomain organization as a putative biological vulnerability underlying suicidality and suggests that biomarkers related to lipid raft function, inflammation, and tryptophan metabolism may improve risk stratification. Moreover, these mechanisms may operate across multiple psychiatric and neurological conditions, supporting a transdiagnostic view of suicide risk. Finally, the proposed framework raises the possibility that interventions targeting these pathways, including modulation of lipid raft composition and dietary supplementation with omega‐3 fatty acids, may represent novel strategies for reducing suicide risk.

Several limitations of this work should be acknowledged. First, the proposed framework is based on the integration of findings from heterogeneous studies, and direct causal relationships remain to be established. Second, many of the reported associations are derived from peripheral biomarkers, which may not fully reflect central nervous system processes. Third, individual variability in biological and psychosocial factors contributing to suicidality is substantial, and the proposed model may not apply uniformly across all populations.

Future studies should determine whether biomarkers related to lipid raft function and tryptophan metabolism can improve risk stratification and whether interventions such as DHA supplementation can modify these pathways in vulnerable individuals.

## Author Contributions

**Lukasz Smigielski:** conceptualization, investigation, writing – review and editing. **Hans O. Kalkman:** conceptualization, investigation, writing – original draft, visualization.

## Funding

The authors have nothing to report.

## Conflicts of Interest

The authors declare no conflicts of interest.

## Data Availability

No new datasets were generated or analyzed in this study.
